# Childhood Psychological Maltreatment and Depression among Chinese Adolescents: Multiple Mediating Roles of Perceived Ostracism and Core Self-Evaluation

**DOI:** 10.3390/ijerph182111283

**Published:** 2021-10-27

**Authors:** Qiong Wang, Ruilin Tu, Wei Hu, Xiao Luo, Fengqing Zhao

**Affiliations:** 1 School of Education, Zhengzhou University, Zhengzhou 450001, China; wangq@zzu.edu.cn (Q.W.); 202012122012109@gs.zzu.edu.cn (R.T.); 202012122012108@gs.zzu.edu.cn (X.L.); 2 PLA Strategy Support Force Information Engineering University, Zhengzhou 450001, China; psyhuwei@163.com

**Keywords:** childhood psychological maltreatment, perceived ostracism, core self-evaluation, depression

## Abstract

Previous studies have primarily focused on the separate mediating role of interpersonal context or personal characteristics in the relationship between childhood psychological maltreatment and depression, neglecting the combined effects, which have limited ecological validity. Therefore, this study investigated the multiple mediating roles of perceived ostracism and core self-evaluation in the relationship between childhood psychological maltreatment and depression. A total of 1592 Chinese adolescents (51.1% boys), ranging in age from 11 to 15 years (*M* = 13.23, *SD* = 0.96), completed a self-report questionnaire regarding demographics, psychological maltreatment, perceived ostracism, core self-evaluation and depression. A multiple mediation model was tested using Model 6 of the PROCESS macro. After controlling for the variables of gender and age, the results indicated that perceived ostracism and core self-evaluation parallelly and sequentially mediated the link between psychological maltreatment and depression. The multiple mediation model could account for 55% of the total effect. In conclusion, the current study helps us better understand the mechanisms of depression caused by psychological maltreatment, and contributes to preventing and intervening in depression among Chinese adolescents.

## 1. Introduction

Adolescents are going through a critical stage when the imbalance in physical and mental development and various growth crises that need to be faced make them prone to emotional problems, especially depression [1]. Depression among adolescents is a major risk factor for suicide and is the second to the third leading cause of death in this age group [2], with more than half of adolescent suicide victims repeatedly suffering from depression [3]. Moreover, depression among adolescents also leads to serious social and educational impairments [4]. In China, which has undergone dramatic social and economic development during recent decades, competition in education and the workplace are getting fiercer, and thus adolescents are under greater pressure to achieve a better life in the future. When these young adolescents enter junior high school, they need to adapt to the environment, establish harmonious relationships and deal with academic workloads [5]. Thus, the pressures placed on Chinese adolescents are considerable and the pooled prevalence rate of depression has reached 24.3% among Chinese adolescents and the number is still climbing [6,7]. Moreover, influenced by Chinese culture which considers academic achievement to be linked with financial success and social status, Chinese people pay more attention to adolescents’ academic performance, relatively neglecting adolescents’ mental health problems, such as depression. Therefore, exploring the determinants and psychological mechanisms underlying depression to reduce the incidence of depression among Chinese adolescents is especially pressing.

Psychological maltreatment in childhood is one of the stressful life events experienced by adolescents and has been increasingly recognized as the core issue in all forms of childhood maltreatment. Recent research has indicated that psychological maltreatment is associated with depression [8], and a growing body of literature has aimed to explore the psychological mechanism by which psychological maltreatment affects depression. However, this research has primarily focused on the separate mediating role of the interpersonal context such as peer attachment [9], or personal characteristics such as self-esteem and mental resilience [10,11], neglecting the combined effects of personal characteristics and interpersonal context. In addition, the cumulative risk model and the ecological systems theory emphasize that the development of individuals is the result of the interaction of various individual factors, interpersonal processes and distal contextual factors. Different risk factors are not independent, but synergistic and interactive [12,13]. Therefore, the current study used a multiple mediation model to explore the mediating roles of perceived ostracism (an interpersonal factor) and core self-evaluation (an individual factor) in the relationship between psychological maltreatment (a family contextual factor) and depression among Chinese adolescents.

### 1.1. Psychological Maltreatment and Adolescent Depression

Psychological maltreatment refers to the harmful and inappropriate parenting behaviors of caregivers that express to children they are unloved, unwanted, worthless or only of value when meeting the needs of others [14], but does not involve physical and sexual contact [15]. It also includes omission and commission acts such as spurning, terrorizing, isolating, exploiting, denying emotional responsiveness and neglecting mental health or education [16]. Psychological maltreatment in childhood can seriously harm adolescents’ mental health, leading to a high risk of symptoms such as depression, anxiety, psychiatric disorders, suicidal ideation and behavioral disorders [17,18]. The negative effect of psychological maltreatment on adolescents’ depressed mood has been well documented. For example, some cross-sectional studies have shown that adolescents who have experienced psychological maltreatment are more likely to be depressed and to report a higher rate of depression than those who have not [10,19,20]. Besides, longitudinal research has indicated that psychological maltreatment may be harmful to the development of adolescent depressive symptoms [8]. Cognitive neuroscience research has also shown that childhood maltreatment is negatively correlated with the volume of brain structures such as the prefrontal cortex and the amygdala, which is consistent with the volume of brain structures being changed in depressed patients [21,22].

### 1.2. The Mediating Role of Perceived Ostracism

Ostracism is defined as “ignoring and excluding individuals or groups by individuals or groups” [23]. The Rejection Sensitivity (RS) Model [24] states that previous rejection imparted by valued others can cause peer rejection, and the perceptions of rejection will elicit cognitive-affective reactions. The extent of individuals’ perceptions of rejection may be inaccurate, but their negative responses to other ambiguous behaviors can cause an actual rejection experience and a self-fulfilling prophecy [25]. According to the RS model, adolescents who have experienced psychological maltreatment may perceive their parents’ behavior as neglect or rejection of them, leading to perceived ostracism. Moreover, anxious expectations about ostracism foster heightened vigilance against ostracism, so that even harmless social interactions are seen as signs of deliberate ostracism, producing experiences of ostracism. Empirical studies support this notion. For example, a cross-sectional study has shown that domestic physical abuse and emotional abuse are risk factors of social peer rejection [26]. Research has also indicated that teenagers perceive parent phubbing as a kind of rejection or neglect of themselves, thus perceiving a sense of ostracism [27].

When individuals treat harmless social interaction as sign of deliberate ostracism, this cognitive predisposition makes them perceive more ostracism. In addition, adolescents who have experienced ostracism would not feel part of their in-groups, although these groups could provide them with belonging and security. Thus, they would be more prone to depression. Empirical evidence is in support of this notion. For instance, some cross-sectional studies have indicated that perceived ostracism is positively related with psychological distress and depression [28,29]. Besides, longitudinal research has indicated that ostracism often precedes higher levels of depression and not vice versa [30]. Combined, perceived ostracism may mediate the association between psychological maltreatment and depression. Although empirical research has not yet directly examined the mediating role of perceived ostracism between psychological maltreatment and depression, cross-sectional studies have indicated that perceived rejection mediates the relationship between workgroup mistreatment and affective outcomes such as depression [31,32]. This suggested that maltreatment would contribute to feelings of ostracism, which, in turn, induce depression. Based on the theoretical and empirical evidence presented above, we put forward the following hypothesis:

**Hypothesis** **1** **(H1).** *Perceived ostracism will mediate the relationship between psychological maltreatment and depression. To be specific, psychological maltreatment will be positively related to perceived ostracism, which will, in turn, make individuals more prone to depression.*

### 1.3. The Mediating Role of Core Self-Evaluation

Core self-evaluation refers to one’s fundamental appraisals regarding self-worth and ability [33], which is a high-order and stable personality trait that will remain consistent over time. Core self-evaluation is also a broad personality, indicated by four more specific traits: self-esteem, generalized self-efficacy, locus of control and emotional stability (low neuroticism) [34]. The self-verification theory holds that people constantly elicit or seek feedback consistent with their self-concept, aiming to maintain and reinforce their original self-concept [35]. Therefore, low core self-evaluators may seek negative feedback and tend to interpret information more negatively, which, in turn, develops a range of negative emotions, leading to depression. Empirical studies have also shown that people with low core self-evaluation are prone to depression, while those with positive core self-evaluation are less likely to experience depression [36,37,38].

Based on attachment theory, a child’s mental representation of the self develops from the early experience of interacting with primary caregivers [39]. Specifically, secure attachment in childhood leads to a positive view of the self, as warm and responsive parenting is correlated with positive self- and other-evaluative core beliefs [40]. However, child maltreatment often undermines people’s attachment security [41], which, in turn, leads to the formation of negative core self-evaluation. Besides, one of the vital developmental tasks of adolescents is gradually completing their own identity and evaluation [42], and the primary caregivers, usually parents, play a crucial role in the development of adolescents [43]. For adolescents who have experienced psychological maltreatment, critical and humiliating comments, as well as the considerable lack of positive feedback from parents, may lead them to doubt their own abilities and a tendency to identify with their parents’ criticism and put-downs of them. Thus, a negative self-evaluation has formed. In short, psychological maltreatment may be an important predictor of adolescents’ core self-evaluation. In addition, empirical studies regarding self-esteem, the specific trait in core self-evaluation, have also provided evidence for the association between psychological maltreatment and adolescent core self-evaluation [44]. For instance, previous research has indicated a strong positive correlation between psychological maltreatment and low self-esteem [17,45]. Combined, core self-evaluation may mediate the relationship between psychological maltreatment and depression. Although no research to date has directly examined the mediating role of core self-evaluation in the relationship between childhood psychological maltreatment and depression, a few studies have shown the mediating role of self-esteem between child maltreatment and depression. For example, a relevant cross-sectional study has shown that child maltreatment can influence depressive symptomatology among adolescents in out-of-home care through the mediating role of self-esteem [11], and a longitudinal study has indicated that self-esteem has a longitudinal mediation effect on the relationship between child maltreatment and early adolescent depression [9]. Based on the theoretical and empirical evidence presented above, we put forward the following hypothesis:

**Hypothesis** **2** **(H2).** *Core self-evaluation will mediate the relationship between psychological maltreatment and depression. To be specific, psychological maltreatment will be negatively related to core self-evaluation, which will, in turn, make individuals more prone to depression.*

### 1.4. The Mediating Roles of Perceived Ostracism and Core Self-Evaluation

Adolescents’ core self-evaluation may be influenced by perceived ostracism. On one hand, the temporal need–threat model of ostracism points out that the sense of neglect and rejection experienced by people during socialization often threatens their own value beliefs and sense of existence, thus causing a decline in self-esteem [46]. On the other hand, empirical research has indicated that ostracism is an important risk factor for self-esteem, and the more severe the ostracism, the greater the threat to self-esteem [47,48,49]. Therefore, we hypothesized that individuals who experience feelings of ostracism might have low core self-evaluation. To sum up, it is possible that psychological maltreatment can influence depression through serial mediation of perceived ostracism and core self-evaluation. As previously discussed, the relationship between psychological maltreatment and depression could be mediated by perceived ostracism and core self-evaluation in parallel. Thus, we expect a multiple mediation model and put forward the following hypothesis:

**Hypothesis** **3** **(H3).** *Perceived ostracism and core self-evaluation will sequentially mediate the relationship between psychological maltreatment and depression. To be specific, psychological maltreatment will be positively related to perceived ostracism, which might be negatively related to core self-evaluation, finally making individuals more prone to depression.*

### 1.5. The Present Study

**Figure 1:**
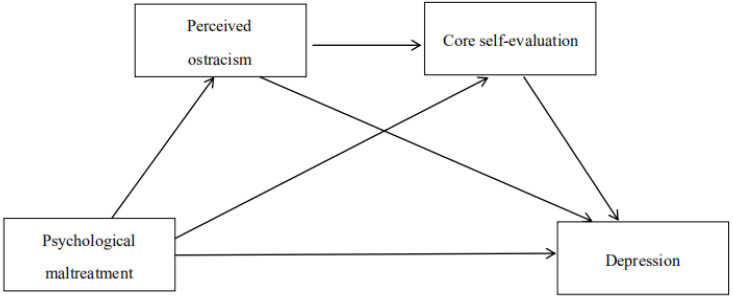
A multiple mediation model of the association between psychological maltreatment and depression.

In this study, we tested a model of the process by which psychological maltreatment could be positively related to adolescent depression. The purpose of the current study was to have a better understanding of the mediating roles of perceived ostracism and core self-evaluation in the relationship between psychological maltreatment and depression among Chinese adolescents. To be specific, the aims of this study were to test the following three questions: (1) to test whether perceived ostracism mediates the relationship between psychological maltreatment and adolescent depression, (2) to test whether core self-evaluation mediates the relationship between psychological maltreatment and adolescent depression, and (3) to test whether perceived ostracism and core self-evaluation mediate the relationship between psychological maltreatment and adolescent depression sequentially and in parallel. Figure 1 shows the proposed multiple mediation model.

## 2. Methods

### 2.1. Participants

In this study, the participants were recruited from a middle school in Henan Province located in the center of China using cluster random sampling. We handed out the questionnaires to all classes and, in total, 1680 questionnaires were obtained. After the invalid questionnaires were discarded, 1592 valid questionnaires were collected, and the valid response rate was 94.71%. The participants consisted of 779 girls (48.9%) and 813 boys (51.1%); 623 (39.1%) were in Grade 7, 527 (33.1%) were in Grade 8 and 442 (27.8%) were in Grade 9. The adolescents’ age ranged from 11 to 15 (*M* = 13.23, *SD* = 0.96).

### 2.2. Measures

#### 2.2.1. Psychological Maltreatment

The severity of child psychological abuse was measured by the Chinese version of the Childhood Psychological Maltreatment Scale [50]. Respondents needed to rate 23 items (e.g., “My parents scold me for no reason”) on a 5-point Likert scale ranging from 1 (never) to 5 (always). The final score was the average of the item scores, with higher scores indicating high levels of psychological maltreatment. This measure has demonstrated good reliability among Chinese adolescents [15]. For the present study, the measure demonstrated good reliability (Cronbach’s α = 0.91).

#### 2.2.2. Perceived Ostracism

An individual’s perceptions concerning ostracism were assessed by the Chinese version of the Ostracism Experience Scale for Adolescents [51]. The scale comprised 11 items (e.g., “Normally, other people seem to ignore me”) and included two sub-dimensions (exclusion and being ignored). The participants rated each item on a 5-point scale ranging from 1 (strongly disagree) to 5 (strongly agree). Among them, the 6th, 7th, 8th, 9th, 10th and 11th items were scored in reverse. The final score was the average of the item scores, with higher scores representing higher perceptions of ostracism. This measure has demonstrated good reliability among Chinese adolescents [52]. For the current study, the measure demonstrated good reliability (Cronbach’s α = 0.89).

#### 2.2.3. Core Self-Evaluation

The Core Self-Evaluation Scale (CSES) was developed by Judge and Thoresen [53] and this research used the Chinese version of the scale. The scale consisted of 12 items (e.g., “On the whole, I am satisfied with myself”). Each item was rated on a 5-point scale ranging from 1 (strongly disagree) to 5 (strongly agree). The final score was the average of the item scores and a high total score indicated high levels of core self-evaluation. This measure has demonstrated good reliability among Chinese adolescents [54]. For the present study, the measure demonstrated good reliability (Cronbach’s α = 0.89).

#### 2.2.4. Depression

The study used the 10-item Chinese short version of the Center for Epidemiologic Studies Depression Scale (CES-D) to measure the severity of depression among the participants [55]. It consists of 10 items (e.g., “I feel very depressed”). Respondents were asked to rate each item in terms of the frequency that each mood or symptom occurred on a 4-point scale (0 = less than 1 day, 1 = 1–2 days, 2 = 3–4 days, 3 = 5–7 days) over the preceding 7 days. Among them, the 5th and 8th items were scored in reverse. The final score was the average of the item scores, with higher scores indicating more severe depressive symptoms. This measure has demonstrated good reliability among Chinese adolescents [56]. For the current study, the measure demonstrated good reliability (Cronbach’s α = 0.87).

### 2.3. Procedure

The research design was approved by the Ethics Committee of the authors’ institution. Written informed consent was obtained from the legal guardians, and oral consent was obtained from school administrators and teachers. We also told the adolescents that they were invited to participate in this investigation voluntarily and could withdrawal at any time. Research assistants handed out the questionnaires to all students with the help of the teacher in the classroom during regular class time. Students completed questionnaires regarding demographics, psychological maltreatment, perceived ostracism, core self-evaluation and depression.

### 2.4. Data Analysis

We used SPSS 24.0 (IBM, Armonk, NY, USA) and the Hayes SPSS macro program PROCESS [57] to organize and analyze the data. First, the descriptive information and correlation matrix were collected. Second, we selected Model 6 to examine the multiple mediating models of perceived ostracism and core self-evaluation in the relationship between psychological maltreatment and depression. Furthermore, the bootstrapping method (5000 bootstrap samples) with 95% bias-corrected confidence intervals was used to test the significances of the total, direct and indirect effects [57]. Confidence intervals that did not include zero indicated that the effects were significant. Before formal data processing, all variables were standardized.

## 3. Results

### 3.1. Preliminary Analyses

The skewness scores of psychological maltreatment, perceived ostracism, core self-evaluation and depression ranged from −0.04 to 1.55, and the kurtosis scores ranged from −0.26 to 3.55. Therefore, the skewness and kurtosis fell within the acceptable range [58].

The means, standard deviations and zero correlations for all study variables are presented in Table 1. As expected, psychological maltreatment was negatively correlated with core self-evaluation (*r* = −0.44, *p* < 0. 001) and positively correlated with perceived ostracism and depression (*r* = 0.28, *p* < 0. 001; *r* = 0.50, *p* < 0. 001). In addition, perceived ostracism was negatively correlated with core self-evaluation and positively correlated with depression (*r* = −0.48, *p* < 0. 001; *r* = 0.47, *p* < 0. 001). Moreover, core self-evaluation was negatively correlated with depression (*r* = −0.69, *p* < 0. 001). The findings of the correlational analysis supported the hypothesis, allowing the mediating effects among variables to be tested further.

### 3.2. Multiple Mediating Analysis

When depression was considered to be the dependent variable, the collinearity analysis of psychological maltreatment, core self-evaluation and perceived ostracism showed that the tolerance values were above 0.65 and that the variance inflation factor (VIF) values were below 1.50. This meant that the variables of psychological maltreatment, perceived ostracism and core self-evaluation had no collinearity issues, which indicated that the results of the mediation analysis were unbiased.

**Figure 2:**
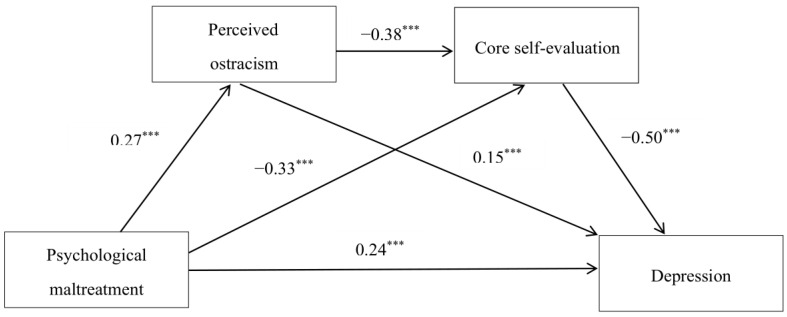
Results of the multiple mediation analysis for depression. *** *p* < 0.001. *N* = 1592.

After controlling for the variables of gender and age, a multiple mediating analysis was conducted to explore the mediating effects of perceived ostracism and core self-evaluation using Model 6 of the PROCESS macro. The results of the multiple mediation analysis indicating the relationships among the variables are presented in Table 2 and Figure 2. Standardized regression coefficients showed that psychological maltreatment was positively related to perceived ostracism (*β* = 0.27, *p* < 0.001), which, in turn, was positively related to depression (*β* = 0.15, *p* < 0.001). Moreover, psychological maltreatment negatively predicted core self-evaluation (*β* = −0.33, *p* < 0.001), and core self-evaluation, in turn, was a negative predictor of depression (*β* = −0.50, *p* < 0.001). In addition, perceived ostracism was negatively related to core self-evaluation (*β* = −0.38, *p* < 0.001).

Table 3 shows the results of the bootstrap analysis. The 95% confidence intervals of each path coefficient did not contain zero, which indicated that the total effects, direct effects and indirect effects were all significant. The multiple mediation effects could account for 55% of the total effects. Specifically, the effect of the path “psychological maltreatment→perceived ostracism→depression” was 0.042, accounting for 8.4% of the total effects; the effect of the path “psychological maltreatment→core self-evaluation→depression” was 0.168, accounting for 33.6% of the total effects; the effect of the path “psychological maltreatment→perceived ostracism→core self-evaluation→depression” was 0.052, accounting for 10.4% of the total effects. In a word, perceived ostracism and core self-evaluation mediated the link between psychological maltreatment and depression sequentially and in parallel.

## 4. Discussion

Research has found that child maltreatment, particularly psychological maltreatment (emotional abuse and/or neglect), is related to a wide range of long-term adverse health and developmental outcomes [59]. Emotional/psychological maltreatment has the strongest association with depression in comparison with other types of child maltreatment [60]. However, psychological maltreatment is the least addressed in child protection and social policy [61]. Therefore, the present study investigated the relationship between psychological maltreatment and depression, and further explored the mechanism of how psychological maltreatment influences depression among Chinese adolescents. As expected, the results supported that perceived ostracism and core self-evaluation serve as mediators, both in parallel and sequentially, on the effect of psychological maltreatment on depression among Chinese adolescents.

### 4.1. The Mediating Role of Perceived Ostracism

The results of the present study were in support of Hypothesis 1, showing that perceived ostracism was an important mediator in the relationship between psychological maltreatment and depression among Chinese adolescents. Specifically, adolescents who were exposed to high levels of psychological maltreatment are more likely to experience greater feelings of ostracism, which may increase the risk of depression. Therefore, perceived ostracism could be one of the explanatory mechanisms for why adolescents with a high level of psychological maltreatment are more likely to be depressed.

In addition to the overall mediation result, each of the separate links in this mediation model is noteworthy. For the first link of the mediation stage (i.e., psychological maltreatment→perceived ostracism), this research indicated that psychological maltreatment has a positive link with perceived ostracism. The result was consistent with previous research indicating that child abuse by parents leads to feelings of rejection [26] and might be partly due to the following reasons. First, individuals who have experienced psychological maltreatment in childhood may perceive their parents’ behavior as rejection and neglect of them, which may sensitize adolescents to readily expect and perceive rejection from others, so they are prone to perceiving others’ harmless actions as ostracism [62] and to experience feelings of ostracism. Second, childhood psychological maltreatment (e.g., parental neglect of a child’s needs) may impair the fulfillment of human’s need to belong [63], making it difficult for adolescents to find a sense of belonging in a group and they thus feel ostracism. Therefore, it is reasonable to find that psychological maltreatment was positively related to perceived ostracism. For the second part of the mediation stage (i.e., perceived ostracism→depression), our findings suggested that perceived ostracism has a positive link with depression. This is in line with previous research showing that perceived ostracism was positively related to depression [28]. According to the basic psychological need theory, ostracism thwarts people’s fundamental needs for social interactions and belonging, which are vital to mental health [64]. Thus, individuals with perceived ostracism may have low belonging and poor interpersonal relationships, making the risk of depression increase. Moreover, social exclusion will activate the anterior cingulate cortex, causing the individual to produce “social pain” similar to physical pain [65], which, in turn, may induce negative emotions such as depression. Therefore, it is reasonable to find that perceive ostracism was positively related to depression. As discussed above, perceived ostracism was not only an outcome influenced by psychological maltreatment but also a sign of the onset of depression.

### 4.2. The Mediating Role of Core Self-Evaluation

In line with Hypothesis 2, our results indicated that core self-evaluation had a significant mediating effect on the relationship between psychological maltreatment and depression among Chinese adolescents. To be specific, adolescents who were exposed to high levels of psychological maltreatment were likely to score lower on core self-evaluation, which may increase the experience of states of depression. Therefore, core self-evaluation could be another explanatory mechanism for why adolescents with high levels of psychological maltreatment are more likely to be depressed. This finding is consistent with previous research that core self-evaluation is a mediator between stressful life events and depression [38].

Similar to the mediation model above, each of the separate links in the mediation model is also noteworthy. For the first part of the mediation stage (i.e., psychological maltreatment→core self-evaluation), this study revealed that psychological maltreatment may be negatively related to core self-evaluation. The result from the present study was consistent with previous research showing that adolescents who have experienced psychological maltreatment generally have low self-esteem [45]. The attachment theory [39] can explain this finding. A good relationship with primary caregivers in childhood will prompt individuals to form a secure attachment and easily obtain a sense of security, which can have many positive effects, such as contributing to mental health and improving positive evaluations of self and others [66]. However, individuals who have experienced psychological maltreatment have difficulty forming secure attachment, so they develop negative core self-evaluation. Moreover, positive and encouraging feedback from caregivers is the main reason for forming positive self-evaluation, while adolescents who are psychologically maltreated may internalize the critical and demeaning messages that their caregivers express to them, and come to the conclusion that maltreatment is deserved due to their negative characteristics [67]. Therefore, it is reasonable to find that psychological maltreatment was negatively related to core self-evaluation. For the second part of the mediation stage (i.e., core self-evaluation→depression), our findings provide evidence for the notion that core self-evaluation may be negatively related to adolescent depression. This is consistent with previous research indicating that adolescent depressive symptoms are related to negative self-evaluation [37]. Adolescents who score lower on core self-evaluation may seek negative feedback from the environment to verify their negative self-concept. They lack confidence in their abilities, adopt negative strategies to cope with life events, and do not take the initiative to deal with their negative emotions, which may lead to increases in anxiety and depression [68]. Therefore, it is reasonable to assume that individuals with a negative view of self may form negative appraisals toward their self-worth and abilities that predispose them to being depressed. As discussed above, core self-evaluation is not only an outcome impacted by psychological maltreatment but is also a cause of depression.

### 4.3. The Mediating Roles of Perceived Ostracism and Core Self-Evaluation

The results indicated that perceived ostracism and core self-evaluation mediated the relationship between psychological maltreatment and depression sequentially among Chinese adolescents, which supported Hypothesis 3. It is noteworthy that perceived ostracism has a negative association with core self-evaluation, which is consistent with previous research [48]. In the adolescent stage, core self-evaluation has greater contextual and external dependence [69]. Specifically, positive life events can provide important information to adolescents for shaping positive core self-evaluations, while a sense of neglect and exclusion or ostracism provide negative evaluation information. Adolescents may internalize this negative evaluation as part of their self-concept, leading to a decreased sense of self-worth and meaning. Thus, a low core self-evaluation has formed.

The aforementioned results demonstrated that psychological maltreatment can also have an effect on depression among Chinese adolescents through parallel and serial mediation by perceived ostracism and core self-evaluation. In addition, the direct effect of psychological maltreatment on depression displayed that psychological maltreatment, as a risk factor, may increase the possibility of depression. This is in support of previous research showing that psychological maltreatment was positively associated with adolescent depression [70].

### 4.4. Limitations and Future Directions

Our research has several limitations that should be acknowledged. First, this study was a cross-sectional design, which means we cannot draw a causal conclusion from the results. Thus, future studies could adopt experimental or longitudinal designs to explore the causal relationship among the variables. Second, we collected the data using self-reported questionnaires only. Although the validity and reliability of these measures have been well established, response bias or socially desirable responses may exist in the present study. Therefore, future studies can collect data from multiple informants (e.g., peers, parents or teachers) to enhance measurement accuracy and replicate our results. Third, the participants of this study were all Chinese adolescent students, so the cross-cultural applicability of the results is limited. Future studies can collect data from different groups to validate our results. Finally, this study only tested the mediating roles of perceived ostracism and core self-evaluation in the link between psychological maltreatment and depression. Exploring other important mediators and underlying mechanisms in the relationship between psychological maltreatment and depression is very important for future research.

### 4.5. Implications

The results of the present study have important theoretical and practical implications. Theoretically, this study using a multiple mediation model may help us understand the mechanisms of depression caused by psychological maltreatment. It also demonstrated that perceived ostracism and core self-evaluation are key factors for adolescent depression. Therefore, this study has deepened and expanded our understanding of the relationship between psychological maltreatment and depression. Additionally, the current study supported the cumulative risk model as well as ecological systems theory, and emphasized that depression is the result of the interaction among interpersonal processes (e.g., perceived ostracism), various individual factors (e.g., core self-evaluation) and distal contextual factors (e.g., psychological maltreatment). Practically, the results contribute to preventing and intervening in adolescent depression. First, given that psychological maltreatment has a positive association with depression, it is necessary to attach great importance to the family context and use appropriate interventions during childhood to reduce rates of adolescent depression. Second, individuals with a low level of core self-evaluation or a high level of perceived ostracism should be given interventional measures to reduce adolescent depression risk. For example, cognitive therapy can help individuals reduce irrational beliefs and change maladaptive schemas, so that they view themselves in a positive manner and do not view the non-hostile behavior of others as ostracism. Ultimately, the risk of depression will be decreased. Furthermore, simultaneous multiple interventions as described above would be more powerful.

## 5. Conclusions

In summary, the present study, which used a multiple mediation model, demonstrated that psychological maltreatment may not only influence depression directly but may also exert an indirect influence on depression through perceived ostracism and core self-evaluation sequentially and in parallel among Chinese adolescents.

## Figures and Tables

**Table 1:** Descriptive statistics and correlations among variables.

Variables	*M*	*SD*	1	2	3	4	5	6
1 Gender	0.49	0.50	1					
2 Age	13.23	0.96	−0.03	1				
3 Psychological maltreatment	1.67	0.55	−0.06 *	0.07 **	1			
4 Perceived ostracism	2.54	0.69	−0.01	0.12 ***	0.28 ***	1		
5 Core self-evaluation	3.61	0.69	−0.06 *	−0.13 ***	−0.44 ***	−0.48 ***	1	
6 Depression	0.70	0.53	0.09 ***	0.14 ***	0.50 ***	0.47 ***	−0.69 ***	1

Note. *N* = 1592. Gender is a dummy variable (male = 0 and female = 1); * *p* < 0.05, ** *p* < 0.01, *** *p* < 0.001.

**Table 2:** Results of the multiple mediation analysis.

Regression Model Outcome Variable	Predictor Variable	Goodness-of-Fit Indices			Regression Coefficient and Significance	
		*R*	*R* ^2^	*F*	*β*	*T*
Perceived ostracism		0.29	0.09	50.24 ***		
	Gender				0.03	0.55
	Age				0.10	4.05 ***
	Psychological maltreatment				0.27	11.29 ***
Core self-evaluation		0.59	0.34	207.65 ***		
	Gender				−0.18	−4.33 ***
	Age				−0.07	−3.29 **
	Psychological maltreatment				−0.33	−15.77 ***
	Perceived ostracism				− 0.38	−17.83 ***
Depression		0.74	0.55	387.17 ***		
	Gender				0.15	4.33 ***
	Age				0.04	2.34 *
	Psychological maltreatment				0.24	12.59 ***
	Perceived ostracism				0.15	7.96 ***
	Core self-evaluation				−0.50	−24.16 ***

Note. * *p* < 0.05, ** *p* < 0.01, *** *p* < 0.001.

**Table 3:** Bootstrap analysis of multiple mediation effects.

Effect	Effect Size	SE	Percentage of Total Effects	95 % CI	
				Lower	Upper
Total effects	0.500	0.021	100%	0.458	0.542
Direct effects	0.238	0.019	47.60%	0.201	0.275
Total mediation effects	0.262	0.019	52.40%	0.227	0.299
Psychological maltreatment→perceived ostracism→depression	0.042	0.007	8.40%	0.028	0.057
Psychological maltreatment→core self-evaluation→depression	0.168	0.014	33.60%	0.142	0.197
Psychological maltreatment→perceived ostracism→core self-evaluation→depression	0.052	0.007	10.40%	0.040	0.065

Note. *N* = 1592. Bootstrap = 5000. → = unidirectional path.

## Data Availability

The data presented in this study are available on request from the corresponding author.
